# Who Reads the Trade Marks Register?

**DOI:** 10.1093/ojls/gqaf001

**Published:** 2025-02-04

**Authors:** Robert Burrell, Michael Handler

**Keywords:** nature of trade mark registration, trade mark specifications, Nice classification, hypothetical trader standard

## Abstract

This article starts with a question that looks like it has been taken from an introductory legal reasoning class, namely, is coffee a non-alcoholic beverage? It will be seen that from a trade mark perspective there is reason to conclude that coffee is definitely a non-alcoholic beverage in Australia, is definitely not a non-alcoholic beverage under the European trade mark regime and may or may not be such a beverage in the UK. This divergence is itself worthy of attention, given efforts to facilitate cross-border registration using common terminology. More importantly, however, this article argues that the reason why trade mark law struggles with questions of this type is because it has never taken a clear view of the person at whom the trade marks register is aimed—in particular, it has never been clear as to the level of expertise and knowledge of the internal workings of the trade mark system that is to be attributed to the notional reader of the register. This oversight has important implications for matters that someone new to the field might imagine would have been resolved long ago.

## 1. Introduction

Is coffee a non-alcoholic beverage? If this were a question in a consumer survey, a clear consensus might well emerge. But for those with legal training, this question is more likely to produce wariness, at least once told they are dealing with this issue as a matter of law. A legal audience will be mindful that a range of interpretive techniques can be brought to bear on questions of this type. Legal education teaches that even the clearest of categories can permit exceptions—that a military truck in a public garden may not fall foul of an ordinance prohibiting vehicles in parks if it has been mounted on a pedestal to serve as a war memorial.^1^ The lawyer will therefore want to know more about the context in which the question of whether coffee is a non-alcoholic beverage has arisen. Confronted with an unfamiliar context, the lawyer will also want to know if this or a cognate question has been adjudicated before and, if so, how it was resolved.^2^ This interrogation of previous decisions is, of course, connected to a belief in the importance of ensuring certainty and predictability in decision making—adjudicatory criteria that the lawyer is going to want to bear in mind generally when deciding whether coffee is a non-alcoholic beverage.^3^

In the course of this article, we will see that from a trade mark perspective there is reason to conclude that coffee is definitely a non-alcoholic beverage in Australia, is definitely not a non-alcoholic beverage for the purpose of the pan-European trade mark regime, and may or may not be such a beverage in the UK. This divergence is itself worthy of attention, given that considerable efforts have been made to facilitate cross-border trade mark registration using common terminology.^4^ In this article, however, we suggest that the reason why trade mark law struggles with questions of this type is because it has never taken a clear view of the person at whom the trade marks register is aimed. This oversight has important implications for matters that someone new to the field might imagine would have been resolved long ago. In particular, the absence of a clear vision of who reads the register has created a penumbra of uncertainty about how the information on the register is to be interpreted and has led to the adoption of mutually incompatible interpretive rules.

To explain and develop these ideas, we start in section 2 by looking at how tribunals in Australia, the EU and the UK approach the interpretation of the specification of the goods and services in respect of which the trade mark is registered. It will be seen not merely that this task is approached in different ways in different jurisdictions, but also that within jurisdictions the existence of different approaches has generally not been adequately recognised. The jurisdiction in which one finds the most comprehensive discussion is the UK, but this has not led to the emergence of a clear position on how specifications are to be read.

In section 3, we demonstrate that the approach adopted to the interpretation of specifications is often inconsistent with other interpretive rules used in trade mark law, in particular, rules that determine what the protected sign ‘is’. Consequently, when it comes to reading information on the trade marks register, there is inconsistency within as well as between jurisdictions.

We then argue in section 4 that the idea that the trade marks register can and should be read by the ‘ordinary trader’ has been given undue prominence. Greater attention needs to be paid to ensuring that the scope of trade mark protection is stable, consistent and predictable. This requires taking seriously the administrative rules and processes that govern how trade marks are filed and registered. This would limit the risk of owners constructing their rights in surprising ways during infringement proceedings and of finding creative ways of sidestepping the requirement that a mark be used to remain on the register. A closer investigation of the question of who reads the trade marks register suggests that this person ought to be conceptualised as having particular skills, expertise and understandings of the rules, procedures and idiosyncrasies of the registered trade mark system.

In section 5, we turn to the likely impact of AI tools on the issues under consideration. Intellectual property offices are looking to roll out ‘preclearance tools’ to provide applicants with an automated assessment of whether a conflicting mark is already on the register. Readers familiar with these developments could therefore be forgiven for thinking that, in the future, the answer to the question posed in the title of this article will be straightforward: ‘machines’. However, far from rendering the question posed here otiose, these developments make the issues considered here more pressing. Current projects rest on an aggregation of data derived primarily from decisions of examiners. But examiners are working against a backdrop of unclear and sometimes contradictory rules. If the issues canvassed here are not addressed, there is a danger that our automated decision-making tools will enshrine ways of working and thinking that are unsatisfactory. Trade mark law will, in other words, develop its own variation of the ‘garbage-in, garbage-out’ phenomenon that may prove to be a significant brake on the ability of AI to revolutionise economic activity.

## 2. Of Beverages, Hosepipes and Valves: International Divergence in the Interpretation of Specifications

The process for registering a trade mark is much the same in every major jurisdiction. Applicants have to establish the identity of the would-be owner, and provide a representation of the mark and a specification of the goods and/or services for which registration is being sought. Applicants are also required to group their specified goods and/or services into one or more classes. For this purpose, most countries use the International Classification of Goods and Services established under the Nice Agreement,^5^ which is a multilateral treaty administered by the World Intellectual Property Organization (WIPO).^6^ The Nice Classification is regularly revised, and the current version divides products into 45 classes, consisting of 34 classes for goods and 11 classes for services.^7^ Classifying goods and services in this way is said to enable the administration of the register and facilitate searches for earlier conflicting marks.^8^

It is generally understood that classification is not determinative of the question of whether specified goods or services are similar to other goods or services in assessing conflicts between marks for the purposes of registration or infringement. This is because products that are clearly dissimilar to each other are sometimes grouped within the same class (electronic calculators and fire extinguishers both fall in Class 9), while near identical products are sometimes grouped in different classes (support bandages are in Class 10; bandages for dressings are in Class 5). One therefore finds long-standing judicial statements and more recent legislative provisions that establish that classification is not determinative of questions of similarity.^9^ Moreover, the Nice Agreement itself makes clear that classification shall not bind member states in how they undertake assessments of similarity.^10^ None of this, however, answers a critical, anterior question—does one take the Nice Classification into account when interpreting descriptions of goods or services in order to understand what such goods or services ‘are’? This interpretive exercise needs to be undertaken before the assessment of whether such goods or services are similar to other goods or services can even begin.

To understand this issue, it is necessary to say something more about how Nice operates. Each Nice class has a ‘class heading’, which serves to provide an indication of the sorts of products that fall within that class. For example, Class 43 is headed ‘Services for providing food and drink; temporary accommodation’. Sitting below each heading are explanatory notes indicating items that are and are not included in that class, and an indicative list of goods or services, organised alphabetically, that have been deemed to fall within that class. For example, the explanatory note to Class 43 provides that the class includes the ‘rental of meeting rooms’ and, consequently, ‘rental of meeting rooms’ is a listed service within the Nice alphabetical list for that class. Approximately 11,000 goods and services are listed under the 45 classes.^11^ The Nice Classification also contains ‘general remarks’ containing criteria to be applied in determining how a good or a service should be classified if it is not contained within any of the alphabetical lists.^12^

Over and above the Nice lists, intellectual property offices have developed their own databases, consisting in many cases of tens of thousands of additional product descriptions, grouped by Nice class. For example, the European Union Intellectual Property Office (EUIPO) has developed a tool called ‘TMclass’, which provides access to a Harmonised Database that expands on Nice and contains more than 78,000 goods and services identified by the EUIPO and national European offices.^13^ An even more extensive list has been developed by WIPO for the purposes of the Madrid system, this being the principal system used to facilitate the registration of marks across multiple jurisdictions. The Madrid Goods and Services (MGS) list^14^ contains approximately 130,000 designations,^15^ and many countries, including Australia, have adopted the MGS list in place of a national list of indicative terms.^16^ By choosing terms approved by the registry in question, applicants can be assured that their specifications will be considered to be acceptable, that is, that an objection will not be raised on the grounds that the description of a good or service is ambiguous or unduly broad. Applicants remain free to describe goods or services using their own terminology, but in so doing run a risk of their applications being delayed or rejected.^17^

The attention that has been given to developing lists of goods and services means that it is almost always possible to find an approved term to describe a product. The effort expended on developing such lists has not, however, resolved all issues of ambiguity. In particular, problems arise where the natural meaning of an approved term would seem to cover goods or services that fall within another class. Nice tends to divide goods according to either their function or their composition. By way of illustration, ‘vehicles’ fall in Class 12 and ‘agricultural machinery’ falls in Class 7. How, then, is a tractor to be classified? The convoluted answer is that most tractors fall in Class 12, but small tractors used for lawn mowing are classified in Class 7. Other examples of difficult borderline cases abound. For instance, ‘milk beverages flavoured with coffee’ fall in Class 29, while ‘coffee beverages with milk’ fall in Class 30; ‘tax planning’ falls in Class 35, whereas ‘financial advisory services related to tax’ fall in Class 36.

It is all too easy to poke fun at some of the results of the current system. There are, moreover, ways of ameliorating some of its problems. The acceptance of a broad term such as ‘clothing’ in Class 25 can be read as encompassing a claim for clothing for protection against fire and chemicals (which falls in Class 9) and protective clothing for medical use (Class 10). However, if offices were to require the use of specific terminology that more closely described the precise goods or services that the applicant intends to provide, fewer problems would arise—narrower claims to, say, items of clothing such as ‘t-shirts’ or ‘headscarves’ are much more self-contained and are less susceptible to being read as encompassing goods that fall within other classes.^18^ Ultimately, however, any classification system inevitably throws up difficult borderline questions. Consequently, even if offices were only prepared to accept relatively narrow terms, difficult questions of interpretation would remain. For example, a narrow claim to ‘armoured land vehicles for military use’ in Class 12 would still raise the question of whether ‘battle tanks’ were in scope—battle tanks falling in Class 13 (weapons).

The inability to solve the problems with which we are concerned by requiring the use of more specific terminology (and the importance of such problems) comes into still sharper focus when we turn to consider a second-order problem of interpretation. This problem relates to how to treat terminology in specifications that appears to cut across sub-headings within a Nice class. Consider a claim to ‘jewellery’ in Class 14, which is headed in relevant part ‘jewellery, precious and semi-precious stones; horological and chronometric instruments’. For the purposes of the Nice system, it is clear that a claim to jewellery would not encompass a claim to watches, since ‘jewellery’ and ‘horological instruments’ form separate class sub-headings. But read according to its ordinary meaning, a claim to jewellery might well encompass watches. A second illustration, and one to which we will have cause to return, is a claim to ‘financial services’ in Class 36. This claim might, when read naturally, extend to ‘insurance services’. But a reading that takes into account the Nice class heading, which is ‘Financial, monetary and banking services; insurance services’, would lead to a different conclusion.

Ultimately, therefore, it is inevitable that ambiguity will sometimes attach to specifications. This raises the fundamental question of how specifications should be read, and what are the interpretive principles that should be employed in undertaking this task. There are two key possibilities. These are that words in a specification should be (i) interpreted by reference to the Nice Classification system or (ii) given their ordinary, dictionary definitions, with no regard being paid to Nice. There are, of course, a number of intermediate positions, but ultimately the question of how much weight, if any, should be given to Nice is critical.

The reason this is of such importance is because the different possibilities implicate different considerations of policy. On the one hand, there is an attraction to the idea that we should apply ‘ordinary’ principles of the construction of documents to trade mark specifications, which would mean giving terms their natural or dictionary definitions. This would accord with the idea that trade mark registration is concerned with ‘practical, everyday realities’.^19^ Judged against these concerns, placing weight on Nice would seem to produce counter-intuitive results. This is because there are times when the ordinary meaning of a word unquestionably includes items that fall into another Nice class or sub-heading: ‘armoured land vehicles for military use’ would naturally extend to ‘battle tanks’. On the other hand, to ignore Nice opens up the potential for abuse on the part of sophisticated actors, because it leads to broader readings of terms in specifications. This provides owners with a wider zone of exclusivity, allowing them to construct their rights in surprising and expansive ways during infringement proceedings and making it easier to rebut allegations of non-use. It also enables owners to draft their specifications in strategic ways so as to make their marks more difficult to find in searches, and it helps them avoid paying higher fees by limiting the number of classes in their applications.^20^

The question of how, and from whose perspective, specifications are to be read has been addressed by senior courts in the jurisdictions with which we are concerned. An analysis of this case law shows not only that fundamentally divergent approaches have been taken, but also that insufficient attention has been given to the normative justifications for the positions being adopted and to the policy factors at stake in determining how specifications should be interpreted.

Australia, after a period of uncertainty, has now moved to the position where words in specifications are to be interpreted by reference to their ‘ordinary’ meanings, guided by how such terms would be understood by other traders, with the Nice Classification playing, at most, a marginal role. This is a consequence of two recent decisions of the Full Court of the Federal Court in *Energy Beverages LLC v Cantarella Bros Pty Ltd*^21^ and *Killer Queen, LLC v Taylor*.^22^

The background to *Energy Beverages* was that Cantarella, a business that sells ground coffee and coffee beans, sought to register MOTHERSKY for goods in Class 30, including ‘coffee’. This application was opposed by Energy Beverages, a seller of energy drinks and the owner of the earlier registered mark MOTHER for goods in Class 32, relevantly including ‘non-alcoholic beverages’. One of the questions before the Court was whether the claim for ‘non-alcoholic beverages’ encompassed ‘coffee’, so that Energy Beverages’ mark would be considered to cover the same goods as in Cantarella’s application. The Court held:

Although for administrative purposes coffee beverages fall to Class 30, it does not follow that a claim to ‘non-alcoholic beverages’ nominated in Class 32 … cannot, as a matter of description, include coffee beverages where such goods are part of a single, broad claim to all non-alcoholic beverages. In any event, the classification of goods and services according to classes is primarily a matter of convenience in administration (for example, in facilitating searches). The nomination of a class for particular goods is not decisive as to the scope of a given registration … The question is whether, *from a business and commercial point of view*, the description ‘non-alcoholic beverages’ … would be understood as including non-alcoholic coffee beverages.^23^

The Court accordingly took into account trade evidence that coffee is sometimes described as a non-alcoholic beverage. Consequently, an article in the June 2008 *Food & Beverage* magazine entitled ‘Non-Alcoholic Beverages Award Finalists’, which referred to a brand of coffee as a finalist, weighed heavily on the outcome of the litigation.^24^ The effect of the Court’s finding that coffee is a non-alcoholic beverage was to short-circuit the inquiry of whether non-alcoholic beverages properly classified in Class 32 were goods ‘similar’ to coffee. The importance of this was reflected in the outcome of the case: at trial, Halley J had held that a claim to non-alcoholic beverages in Class 32 did not encompass coffee, and went on to conclude that the two sets of goods were not ‘similar’, so that he would have allowed Cantarella’s MOTHERSKY application to proceed to registration.^25^

In *Killer Queen*, the relevant issue was whether a specification for ‘clothes’ in Class 25 should be taken to include footwear and headgear. One of the arguments put forward against this interpretation was that the Nice class heading (‘Clothing, footwear, headgear’) could be taken into account to limit the scope of the specification. The Full Court essentially followed the approach it had taken in *Energy Beverages*. It reaffirmed that the register is to be interpreted through the eyes of other traders^26^ and that terms in specifications should be construed as ‘ordinary English words’ unless shown by evidence to have a ‘special meaning to those to whom they are taken to be addressed’.^27^ It therefore agreed with the trial judge’s interpretation of ‘clothes’ as a term whose ‘ordinary meaning’, confirmed by dictionaries, does not encompass footwear or headgear.^28^ On the relevance of Nice, the Court agreed that classification is primarily a matter of administrative convenience and that the nomination of a class is not decisive as to the scope of a specification,^29^ but then made a slight qualification to *Energy Beverages*. Specifically, it indicated that Nice headings could help determine the *ordinary* meaning of the words used in a specification.^30^ This means, for example, that there are ‘circumstances where a class heading confirms the meaning arrived at independently of the class heading itself’.^31^ In other words, Nice provides another source, alongside dictionaries and evidence from the trade, to determine what a term would mean to traders in the field.

In determining that neither the Nice class heading nor the fact that goods or services have been specified in a particular class is decisive as to the scope of a specification, the Full Court in both *Energy Beverages* and *Killer Queen* drew support from the first instance decision in *Nikken Wellness Pty Ltd v van Voorst*.^32^ However, that case arguably dealt with the narrower question of how the words in a specification are to be read in the case of a manifest error in classification. In that case, a mark had been registered for ‘the manufacture of magnets for medical purposes’ (ie a service) in Class 10, which covers goods including medical instruments. Wilcox J accepted the argument that the class number should be ignored, meaning that the mark was treated as being registered in respect of manufacturing services, notwithstanding that these should have been classified in Class 40.^33^ The case did not consider what was meant by ‘the manufacture of magnets for medical purposes’ as a service, and therefore raised quite different policy issues from those at stake in *Energy Beverages* and *Killer Queen*. In addition, the Court in *Killer Queen* did not engage with the fact that earlier first instance decisions where it was held that it is appropriate to take Nice into account when interpreting the words of a specification^34^ were consistent with a longer line of pre-harmonisation UK authority.^35^ Nevertheless, the position in Australia is now clear. Words in a specification are to be given their ordinary dictionary definitions, with further assistance being drawn from how they would be understood in trade. At most, Nice can be used to confirm such understandings.

In marked contrast, in the EU, terms in specifications *are* interpreted with reference to Nice. As the EUIPO’s Trade Mark Guidelines put it, ‘Each term is assessed in the context of the class in which it is applied for’.^36^ To illustrate, the Guidelines give the example of clothing, noting that while ‘clothing’ alone would be classified in Class 25, ‘clothing for protection against fire’ would be classified in Class 9 and ‘clothing for operating rooms’ would be included in Class 10, going on to state:

From these examples it is obvious that the term *clothing* can be interpreted in various ways but must always be defined by purpose or market sector pertaining to a particular Nice class. In addition, it shows that *clothing* in Class 25 would **not** cover any of the [specific] categories of goods mentioned above.^37^

The EUIPO’s position has been approved by the General Court in at least 10 cases.^38^ The General Court’s 2023 decision in *Wenz Kunststoff GmbH & Co KG v EUIPO*^39^ provides a particularly nice illustration of the significance of reading down language in a specification by reference to Nice. Wenz was the registered owner of MOULDPRO for ‘rapid-release couplings for hoses, hose connection couplings’ in Class 17. This class is relevantly headed ‘pipes, tubes and hoses, not of metal’. A third party sought to have Wenz’s registration revoked on the basis that Wenz had not used the mark in relation to the specified goods, which Wenz sought to resist by demonstrating use in relation to metal couplings and metal fittings for hoses. The General Court observed that

the descriptions ‘rapid-release couplings for hoses’ and ‘hose connection couplings’ are, in a purely literal sense, rather broad and could include a large variety of those goods, irrespective of their characteristics and, in particular, of the material of which they consist.^40^

However, it went on to say that ‘the registration of the contested mark must be understood in the light of the Nice Classification’^41^ and, more specifically, that

reference should … be made to it in order to determine, where necessary, the range or the meaning of the goods in respect of which a mark has been registered. In particular, where the description of the goods for which a mark is registered is so general that it may cover very different goods, it is possible to take into account, for the purposes of interpretation or as a precise indication of the designation of the goods, the classes in that classification that the trade mark applicant has chosen.^42^

Taking into account that Class 17 covers only non-metallic goods, while metal goods that cannot be classified in other classes according to their function or purpose would be classified in Class 6, the General Court found that Wenz’s specification was limited to ‘non-metallic’ couplings. The effect of this was that Wenz’s registration was revoked for non-use.

The position in the EU is thus that Nice is to be taken into account in interpreting specifications. This is subject to two qualifications. First, in cases where there has been a manifest error in the classification of goods or services, there is scope to set the classification aside. This is a position the General Court has adopted in at least two decisions.^43^ Second, there may be situations, albeit rare, where there is genuine uncertainty about the class in which goods or services should be classified. In such cases, the General Court has indicated that the owner should be given the benefit of the doubt. Thus, if there is some uncertainty about whether or not an owner’s goods were properly classified, then a clear demonstration of use would be sufficient to preserve the registration, even in the face of an argument that such use took place in relation to goods falling into a different class.^44^ In section 4, we note that if other jurisdictions were to follow the EU approach, there would also be good reasons for adopting these qualifications.

What is most striking about the EU approach in the normal case, however, is that the General Court has sought to articulate a set of characteristics of ‘readers’ of specifications that marks them out as having a specialist understanding of the operation of the trade mark system. As the Court stated in *Wenz*, building on a statement it first made in its 2014 decision in *DTM Ricambi Srl v OHIM*, the words used in a specification

must be interpreted from a systematic point of view, having regard to the logic and the system inherent in the Nice Classification, while taking into account the [class headings] and explanatory notes, which are relevant in determining the nature and purpose of the goods in question.^45^

We are supportive of the EU position. Missing, however, from both the EUIPO’s Guidelines and the case law is a clear articulation of the normative justification for the approach being adopted—the desirability of using Nice as an interpretive aid is treated as self-evident. Importantly, moreover, the use of Nice in this way is arguably inconsistent with other aspects of EU trade mark law. Before exploring these issues, we turn to consider the position in the UK, where a different approach again has been adopted.

The question of how specifications are to be interpreted has been treated as a live issue in the UK and, as a consequence, has attracted more policy discussion than in Australia or at the EU level. The position adopted looks, on its face, to lie somewhere between the Australian and EU approaches. It is also easy enough to state—namely, that attention can be given to the Nice system to resolve problems of ambiguity. The leading authority remains the Court of Appeal’s 2001 decision in *Reliance Water Controls Ltd v Altecnic Ltd*.^46^ In that case, Altecnic, a maker of valves for hot water systems, sought to apply for registration of CAREMIX for ‘Valves; valves for use in water circulation; blending valves’. These were initially specified in Class 7, but Altecnic sought to have the goods reclassified in Class 11 (which covers thermostatic valves), to which the registry agreed. A competitor, Reliance, opposed Altecnic’s application for registration on the basis that the reclassification was impermissible under section 39 of the Trade Marks Act 1994. That provision permits amendments to applications only to correct ‘obvious mistakes’, and only then with the proviso that the amendment not ‘extend the goods or services covered by the application’. Reliance argued that the reclassification was impermissible both because the mistake was not obvious and, more importantly for present purposes, ‘as it effectively add[ed] goods not covered by the original application’^47^—that is, goods properly classified in Class 11. In holding that the proposed amendment was impermissible, Mummery LJ observed that

the Registrar is entitled to treat the Class number in the application as relevant to the interpretation of the scope of the application, for example, in the case of an ambiguity in the list of the specification of goods. The application is a considered statement of the applicant which, on ordinary principles of the construction of documents, has to be read as whole to determine its meaning and effect. The fact that the internationally agreed Nice Classification System has been devised to ‘serve exclusively administrative purposes’ … does not mean that the selection by the applicant of one or more Class numbers in his application for registration has to be totally ignored in deciding, as a matter of the construction of the application, what the application is for and whether it can properly be amended.^48^

The upshot was that Reliance succeeded and the mark was remitted for re-examination in Class 7, that is, in a form that Altecnic had no obvious interest in pursuing.

The application of *Altecnic* has, unfortunately, proven difficult and the case remains controversial. It is notable, for example, that in *Omega Engineering Inc v Omega SA*, Arnold J (as he was then) queried whether *Altecnic* applied in the infringement context and, if so, what its effect might be.^49^ Even more significantly, his Lordship questioned whether the decision had been correctly decided, noting that in overturning Laddie J at trial the Court of Appeal had departed from ‘a judge of great experience in this field’ and that the House of Lords had granted leave to appeal before the case was settled.^50^ Leaving aside this last-mentioned concern, significant uncertainty remains about what was intended in *Altecnic*. It is perhaps possible to read the decision broadly, that is, that where a term has been used in a specification that is naturally apt to cover goods or services registrable in multiple classes, reference can be had to the class(es) in which the mark has been registered in interpreting that term. This is the way *Altecnic* was interpreted in *Multi-Access Ltd v Guangzhou Wong Lo Kat Great Health Business Development Co Ltd*.^51^ In that case, one issue was whether the sale of canned liquid herbal tea (tea falling in Class 30) could be considered to involve use in relation to ‘beverages for medicinal purposes’ in Class 32. The court considered that regard could be had to Nice in interpreting specifications even where the words used were clear and precise (returning to the example of ‘valves’ from *Altecnic* as a clear and precise term, but whose scope could only be determined by reference to the class in which the valves had been specified).^52^ On this basis, the court held that there had been no use of the mark in relation to the registered goods.

Returning to the example given in the opening paragraph of this article, *Multi-Access* would seem to suggest that, under UK trade mark law, coffee would not be considered to be a non-alcoholic beverage. But the position is not in fact so clear. This is because other cases have adopted a more limited reading of *Altecnic*. On this more limited view, Nice is only to be used as an interpretive aid where there is some inherent ambiguity in the language used in the specification. The consequences of this more limited interpretation can be seen in *Pathway IP Sarl v Easygroup Ltd*^53^ and *FIL Ltd v Fidelis Underwriting Ltd.*^54^ In *Pathway IP*, the question was how ‘the provision of office facilities’ in Class 35 was to be read. Henry Carr J held that this phrase was sufficiently clear and precise that no reference to Nice was necessary.^55^ As such, the term was held to include such things as the provision of office furniture, even though such services would fall within Class 43. In a similar vein, albeit dealing with Nice sub-headings, the question in *FIL v Fidelis* was whether a claim to ‘financial services’ encompassed ‘insurance services’, despite the fact that these form different sub-headings in Class 36. This question was answered in positive terms, with Arnold J finding that insurance services were ‘within the core of the ordinary meaning of “financial services”’,^56^ based in part on the Wikipedia definition of the latter term.^57^

If the more limited reading of *Altecnic* is correct, it is hard to know when Nice is relevant. There are arguably few terms that are routinely accepted by offices that are inherently ambiguous: those maintaining lists of approved terms are not generally incompetent at what they do. The issue is therefore not one of ambiguity. Rather, it is an issue of scope, clarity and predictability. The uncertainty in the UK position is reflected in similar vagueness as to the characteristics of the person who is interpreting the specification. At times, courts are comfortable falling back on the idea that the reader is a trader interested in ‘how the product is, as a practical matter, regarded for the purposes of trade’,^58^ who would give terms in a specification their ‘ordinary or natural meaning’.^59^ In contrast, in *Multi-Access*, it was accepted that the mechanics of the registration system are such that some understanding of Nice must be ascribed to the person interpreting the specification.^60^ Uncertainty about the identity and characteristics of the hypothetical reader is itself grounded in a lack of clarity as to how the competing policy concerns at play should be assessed and, indeed, to what those competing policy considerations are.

## 3. Of Colours, Scents and Headings: Internal Inconsistencies in Construing Information on the Register

The marked difference in how jurisdictions approach the interpretation of trade mark specifications is notable. It is also a cause for concern when seen against the backdrop of efforts to facilitate transnational trade mark registration. There is also no immediately obvious reason for this divergence—the policy issues are the same from country to country and there is nothing in the legislative arrangements of the jurisdictions being considered here that explains what has occurred. The answer therefore seems to lie in different understandings of who the reader of the trade marks register is: in Australia, the reader is taken to be an ordinary trader; in the EU, the reader is an expert with an in-depth knowledge of the Nice system. On closer inspection, however, this explanation also becomes unconvincing.

The idea that the reader of the register in Australia is the ordinary trader does find a place in other interpretive rules. This idea can be seen, for example, when questions have arisen as to how one should approach the relationship between the representation of the mark that appears on the register and in any accompanying written information. In particular, this issue has arisen in cases involving colour marks, where the question is whether priority should be given to the graphic representation of the mark or to any accompanying description that may, for example, describe the colour by reference to the Pantone system. In the Federal Court’s decision in *Frucor Beverages Ltd v The Coca-Cola Co*,^61^ it was held that it is the graphic representation that determines what the sign ‘is’ and hence the scope of protection. This approach has been firmly tied to the characteristics of the person inspecting the register, it being said:

I do not accept that a person inspecting the Register would approach that task with a mindset that the description is a more precise identifier of the colour that is claimed. Further, I do not accept that a person inspecting the Register should be taken as having an understanding that the reproduction of colour through processes such as scanning, uploading, downloading and printing can be compromised in the process. A person inspecting the Register is entitled to act on the assumption that the trade mark applicant’s own depiction of colour in the representation accompanying the application is accurate.^62^

This approach appears, superficially, to be on all fours with the approach adopted in *Energy Beverages* and *Killer Queen* to the reading of specifications. On closer inspection, however, the matter is less clear-cut. It was seen above that the meaning of ‘non-alcoholic beverages’ was treated as an empirical matter, with evidence of trade usage informing the scope of this term. There is, in contrast, no suggestion that the attributes of the person looking at the sign are to be determined empirically. The court in *Frucor* did not frame the problem in terms of the failure of the trade mark owner to lead evidence as to what the readers of the register would make of the Pantone system. Moreover, if the court had approached the question in that way, then the attributes of the reader would surely change according to the field in question—it is hard to imagine that traders concerned with graphic design services would not understand that the reproduction of colour can be compromised through uploading, scanning, printing and the like. The approach adopted to Pantone references in Australia is therefore probably to be explained by a desire to ensure that the information on the register is self-contained and readily accessible. These are perfectly plausible policy goals, but they are inconsistent with the idea that the meaning of ‘non-alcoholic beverage’ should be determined by reference to extrinsic evidence.

The attributes of the person reading the register in Australia become still more complicated once certain legislative rules are taken into account. *Frucor* proceeds on the basis that a person looking at a colour mark on the register will assume that what they see on the register is the sign that attracts legal protection and that they can (and should) rely on the representation without reference to the accompanying written description. A person consulting the Australian register could not, however, safely adopt this stance in relation to word and device marks that feature colour. This is because section 70 of the Trade Marks Act 1995 (Cth) provides that the default rule is that a mark ‘is taken to be registered for all colours’. The person looking at the register cannot therefore rely on what they see on the register. They can only do so if there is an express endorsement on the register to indicate that colour *is* a feature of the mark, that is, if the accompanying written description tells them that it is safe to do so.^63^

In Australia, one can therefore find interpretive rules that prioritise ensuring that the information on the register is self-contained or that demand specialist knowledge of when and how colour becomes a feature of a mark. These rules are inconsistent with the prevailing approach to interpreting trade mark specifications. This inconsistency has its origins in the different roles performed by the notional ‘ordinary trader’. To explain, Anglo-Australian trade mark law has long taken the position that the register is intended to be read by ordinary traders.^64^ On closer examination, however, although the ‘ordinary trader’ has consistently been called into aid, the interpretation of the information on the register has sometimes been seen through a more policy-oriented lens. In other words, much like trade mark law’s better known ‘notional buyer’ or ‘average consumer’, one finds the ordinary trader being employed as a normative construct as well as a vehicle for taking into account the likely reaction of real market actors.^65^

When one turns to the position in the UK, there is a good argument that the mutable understanding of how information on the register is to be interpreted found in old Commonwealth cases was carried across into early cases under the harmonised European regime.^66^ It arguably continues to inform the current UK approach to reading specifications. Crucially, however, and irrespective of any question of the hangover of old ideas, under the harmonised European regime one finds similar tensions emerging.

In section 2, we saw that the General Court has pushed, particularly in the non-use context, for an approach to interpreting specifications that places a significant degree of weight on Nice. In so doing, it has made it clear that the reader of the register is a person with a detailed understanding of the workings of the trade mark system. This understanding, however, sits uncomfortably with the vision of the Court of Justice of the European Union (CJEU) of the way in which the system ought to operate. Starting with its decision in *Sieckmann v Deutsches Patent- und Markenamt*, the CJEU has emphasised that marks must appear on the register in a form that allows economic operators ‘to receive relevant information about the rights of third parties’^67^ and ‘to determine the precise subject of the protection afforded’.^68^ Given this starting point, the CJEU held that olfactory marks were not capable of clearing the requirement of graphical representation under the original Directive.^69^ The CJEU returned to this idea of the register being designed to convey information to ordinary economic operators in *Chartered Institute of Patent Attorneys v Registrar of Trade Marks*,^70^ a case on the interpretation of specifications. In that case, the applicant had applied to register IP TRANSLATOR in Class 41 in relation to ‘Education; providing of training; entertainment; sporting and cultural activities’, this being the entire heading to Class 41. Whether the mark was distinctive depended on whether the specification was to be treated as extending to ‘translation services’, which fall within Class 41, but which are not represented in the class heading. The CJEU held that the use of class headings in a specification should be read as a claim only to the items set out in that heading and not to all goods or services in the class. Again, the Court justified its approach by prioritising the understanding of the ordinary economic operator,^71^ who, by necessary implication, was taken not to understand the widespread practice of using class headings to claim all goods or services falling in that class.

It might be objected that *Sieckmann* really turned on issues of public policy, above all, the idea that the sign in question ought to be clearly delineated, rather than on how the average economic operator would understand the sign. On this view, any distinction between *Sieckmann* and the *Wenz* line of cases may be illusory. It is, however, more difficult to make a similar claim about *IP Translator*, where the Court seems to have been genuinely motivated by what the ordinary economic operator would understand in reaching its decision. Any attempt to explain *IP Translator* in purely policy terms would be problematic. Requiring applicants to switch from using class headings to using long lists of specific goods and services does little to deal with the potential problems of overclaiming and, indeed, can make it harder to determine whether there is a conflict with earlier marks.^72^

Consequently, whatever its origins might be, the ordinary economic operator has become more than a merely rhetorical device, as arguably can also be seen in the approach adopted to the interpretation of marks depicted on the register in black and white or in greyscale. To explain, prior to 2014, there was a division between national offices in the EU as to how such marks were to be interpreted. Some offices adopted a ‘black and white covers all’ approach (that is, a mark registered in black and white would be treated as extending to representations in any colour). Others applied a ‘what you see is what you get’ approach (that is, a mark registered in black and white would be treated as covering representations in black and white only). In 2014, the precursor to the EUIPO adopted the ‘Common Communication on the Common Practice of the Scope of Protection of Black and White (“B&W”) Marks’, committing participating offices to the ‘what you see is what you get’ approach.^73^ In opting for this approach, the Common Communication took the path of least resistance, since that was already the approach used within the European Office. But even with that caveat in place, there is a good argument that the figure of the ordinary economic operator (who would not be taken to have the expertise to understand the ‘black and white covers all’ approach) helped make the ‘what you see is what you get’ approach the obvious choice.

The tension as regards the attributes of the person who reads the trade marks register may be less immediately obvious in the EU than in Australia, but is nevertheless real. There is more than enough in the explicit and implicit characteristics of the ordinary economic operator to challenge the *Wenz* line when the question of how specifications are to be interpreted finally reaches the CJEU.^74^ For this reason alone, it is by no means inconceivable that the CJEU would sweep away the *Wenz* line. In addition, we have seen that the CJEU has indicated in general terms that Nice serves a purely administrative function.^75^ To this might be added that the manner in which *IP Translator* was codified into the EU Trade Marks Directive and Regulation has resulted in a provision that can perhaps be misread as being inconsistent with the *Wenz* line.^76^

Turning to the UK, it is also not hard to imagine that the tension between the construction of the reader of the register in *Wenz* and the figure of the ordinary economic operator found in *Sieckmann* and *IP Translator* could make it easier for a British court to dismiss the *Wenz* line as being inconsistent with established authority. This is important because early indications are that UK courts are most likely to strike out in their own direction, post-Brexit, in circumstances where there is conflict within EU case law.^77^ There is, therefore, good reason in both the EU and the UK to determine as a normative matter how the interpretation of specifications is to be approached. This forms the focus of the next section, where we argue that the *Wenz* line is correct and suggest that the reader of the register should be conceptualised as an ‘insider’ with a detailed understanding of the workings of the trade mark system.

## 4. Who Should Be Considered to Be the Reader of the Register?

The idea that information on the register is to be interpreted by ordinary traders has intuitive appeal. After all, the trade mark system is clearly designed for the benefit of traders—to ensure that those with an interest in using marks can secure property rights in them and to ensure that other traders have clear notice of those rights, to assist in the smooth functioning of markets. However, there is a problem in simply assuming that the register is capable of providing information of a type that will allow ordinary traders to determine the scope of the rights enjoyed by the registered owner.

Trade mark registers are much less efficient at communicating valuable information to ordinary economic operators than is often assumed. We therefore need to approach with caution judicial pronouncements to the effect that the register allows the public to ‘receive relevant information about the rights of third parties’ and to ‘determine the precise subject of the protection’.^78^ One difficulty is that the governing rules on infringement are such that one needs to have a fairly detailed understanding of trade mark law to make sense of the scope of protection. Infringement extends to the use of ‘similar’ marks, a concept that goes beyond mere visual and aural resemblance and which has been interpreted in many jurisdictions in a manner leading to outcomes that are hard to predict even for seasoned practitioners.^79^ Moreover, infringement can also extend to cases where the defendant’s use is on goods or in relation to services that are dissimilar to those for which the mark is registered. In such cases, the reader would need to look beyond the register (for example, to seek out information about the reputation of the registered mark) to determine whether use of a similar mark in relation to dissimilar goods or services would likely infringe.^80^

The ability of the register to convey meaningful information to the ordinary trader is further constrained by interpretive rules governing how signs and specifications are to be understood. In the preceding section, we noted that Australia has adopted the counter-intuitive position that even where a mark is represented in colour, colour only forms a feature of the mark if there is an express endorsement on the register to this effect. Similarly, although the shift in the UK to the ‘what you see is what you get’ approach for marks represented in black and white sweeps away an interpretive rule only readily comprehensible to trade mark insiders, it is far from clear how tribunals will interpret the scope of black and white marks, particularly those in respect of which applications for registration were made before the Common Communication came into effect.^81^

Turning to specifications, there is scope for asking whether ordinary traders would feel comfortable interpreting the terminology that one finds in lists of approved terms. It is arguable that approved lists steer applicants to employ terminology that abstracts away from the marketplace language more normally used to describe goods and services. To take a fairly straightforward example, how would the ordinary trader who consults the register interpret a claim to ‘cafeteria services’ in Class 43? Would they regard this as a claim limited to the sort of establishment found in factories, schools, colleges and universities, or would it be regarded as extending to any ‘type of food service location in which there is little or no waiting staff table service’?^82^ Even someone versed in trade mark law could be forgiven for being uncertain about the boundaries of this claim. Indeed, if a hypothetical trader were certain that such a term only extends to canteens found in large institutions, they might well be misled as to the scope of protection.

A further issue, and one that goes to the role of Nice as an interpretive device, is that specifications are not always drafted in a self-contained manner. It is still not uncommon to find that the specification itself will be expressly bounded by the relevant Nice category. For new trade marks, this will occur through the use of express qualifications, such as that the specified goods or services are ‘in this class’ or are ‘not included in other classes’. This form of drafting is designed to pre-empt or respond to any concern from an examiner that the goods or services might extend to cover those that ought properly to be classified elsewhere. Although this form of drafting is by no means ubiquitous, it is still encountered relatively frequently. For example, 8488 applications for registration in Australia, filed from 1 September 2020 to 1 September 2024, used the language of ‘in this class’ in their specifications. In the same period, there were 1878 applications containing the term ‘not included in other classes’.^83^ A rather different form of Nice-bounded drafting is to be found in older marks that were filed at a time when it was permissible to claim ‘all goods in this class’. There are, for example, still 2700 marks on the Australian register that use this form of drafting.^84^ When language expressly referring to Nice is still routinely encountered, it makes little sense to oppose using Nice as an interpretive aid on the basis that this would make the system inaccessible to the ordinary trader. This point fell to be considered in *Multi-Access*, where it was concluded that Nice-bounded claiming precludes the specification being interpreted from the standpoint of the ordinary trader or ordinary consumer. As David Stone, sitting as a deputy High Court judge, put it:

‘all included in Class 5’ is not something that can be left to the interpretation of the notional consumer, as the notional consumer will have no idea at all what the expression means. Rather, ‘all included in Class 5’ is to be interpreted by the tribunal by reference to the Nice Classification itself.^85^

Taken together, the above analysis suggests that the register can only ever convey reliable information to traders who have a lawyer on hand to explain what the information on the register means. Seen against this background, there is far less reason to object to attributing a detailed understanding of the workings of the register to the hypothetical reader of the information contained therein. There is also no reason to characterise as problematic administrative practices that facilitate placing information on the register in a form that is only readily understandable by trade mark insiders. There is, for example, no reason to characterise Nice-bounded claiming as something that ‘not infrequently causes[s] difficulty’.^86^ There are, moreover, positive reasons for interpreting the information on the register from the perspective of the expert reader who is taken to understand the rules, procedures and idiosyncrasies of the trade mark system. The most obvious implication of this approach is that concerns about the use of Nice as an interpretive aid could be set aside, and that such use might instead be seen to offer a number of benefits.

A major advantage in using Nice as an interpretive aid is that for trade mark insiders the classification system can help resolve ambiguity about what the boundaries of protection might be. For example, an expert reader will know that a claim for ‘protective work gloves’ in Class 9 will not extend to protective gloves for medical use, since these fall in Class 10. The entire purpose of the register is to provide clear, accessible and reliable information and, for an expert audience, using Nice as an interpretive device can assist considerably. Take, for instance, a person who is conducting a search of the register as part of a ‘clearance’ process, that is, they are searching on behalf of a client to see whether it would be safe to use a proposed mark for particular goods or services. If claims are not to be read as being bound by Nice, then such searches become much more difficult. It would no longer be enough to assure a proposed manufacturer of personal protective equipment for medical use that there are no conflicting marks registered in Class 10 nor in any ‘related’ class.^87^ Rather, it would be necessary to scour the entire register to seek to identify terms in specifications for seemingly unrelated goods that might be construed in such a way as to create an overlap: a claim for ‘protective work gloves’ in Class 9 would have to be identified and assessed.

The process of clearing trade marks for use by market entrants is also associated with the relationship between the Nice Classification and the requirement that registered marks be used. Trade mark law has a ‘use it or lose it’ rule, whereby marks that have not been put to use within a designated period (say, three or five years^88^) are liable to be expunged from the register. This requirement of use is connected to the underlying justifications for the trade mark system and, in particular, to the idea that marks that are not in use are not capable of providing information to consumers about the provenance of goods and services.^89^ The consequence is that market entrants who find that an unused trade mark is barring their way are entitled to have it expunged from the register. This process becomes considerably more fraught, however, if trade mark owners can point to use on goods that would be treated as falling in a different class. To build on the above example, this might, for instance, mean that an owner of a trade mark registered for ‘protective work gloves’ in Class 9 would be able to preserve its registration by pointing to use on protective gloves for medical use.

Importantly, moreover, allowing owners to preserve their marks on the register by pointing to use on goods falling in a different class causes real difficulties in cases where partial non-use is established. Consider, for example, the owner of a mark registered in relation to ‘protective clothing’ in Class 9 who can only point to use on medical gloves. In such a case, the correct approach is to redraw the specification to something that fairly captures the trade in which the owner is actually engaged.^90^ On this example, that might well involve redrawing the specification as ‘protective gloves for medical use’, but if a tribunal were to proceed in this way, this would result in a specification that would clearly and unquestionably relate to goods that ought to have been registered in a different class, courts having no power to order a mark to be reclassified.^91^

The concern that trade mark owners might be able to proffer surprising interpretations of their marks during non-use proceedings is closely connected to the concern that a failure to allow Nice to be used as an interpretive device opens the door to tactical misuse of the trade mark system. One form of misuse, about which there is growing concern, is that the system enables parties to ‘reconstruct’ and expand the scope of protection for their marks. An area where this plays out is in the area of three-dimensional marks that consist of the shape of packaging combined with an inherently distinctive word or device mark. Such marks are likely to sail through examination on the basis that consumers would pay most attention to the distinctive word or device component, but once on the register, there is nothing to prevent an owner threatening to bring a claim for infringement against a party using a similar packaging shape but without a similar word or device.^92^ Another area involves the interpretation of the specification, specifically, where a party to a dispute insists that a term in its specification should be given a surprising or expansive interpretation in an attempt to persuade a tribunal of the existence of a conflict. It is difficult to imagine that the opponent in *Energy Beverages* had coffee in contemplation when it filed for ‘non-alcoholic beverages’: well-advised and resourced international corporations tend to file for all of the specific goods and services in relation to which they have an interest, and usually more besides.^93^ Using Nice as an interpretive aid reduces the danger of expansive *ex post* redefinition of marks.

Another form of misuse involves the strategic misfiling of applications for registration. This involves drafting specifications so that they appear to the registry to cover only goods or services in Class X, even though goods and services in Class Y were always in contemplation. For example, a party interested in traditional skateboards and electric skateboards might only specify ‘skateboards’ in Class 28, even though motorised skateboards fall in Class 12, such that the party ought to have applied in both classes. This might be done for a number of reasons. The party might be trying to reduce its filing fees (in most jurisdictions, such fees are linked to the number of classes in which the application has been filed), although, given the overall small sums involved, this is unlikely to be a weighty consideration for most parties sophisticated enough to engage in tactical misuse of the system. More common reasons for engaging in such behaviour are to avoid conflicts with earlier registered marks and, in the case of trade mark ‘trolls’, to make marks harder to identify until after the point that the party from whom they are seeking to extract a ‘toll’ has committed itself commercially to use of a confusingly similar mark. The success of such strategies is, however, contingent on Nice not being used as an aid to interpretation: it only makes sense for the manufacturer of electric skateboards (or the electric skateboard troll) to file for skateboards simpliciter if there is some prospect of persuading a tribunal down the line that electric skateboards fall within the language used.

Thus far, we have seen that allowing Nice to be used as an interpretive device can be an important mechanism generating clear and predictable boundaries for the benefit of potential defendants and those seeking to challenge registrations. There are, however, circumstances where a failure to make reference to Nice might cause injustice for trade mark owners. *Acetest*, an old UK decision, helps illustrate the point. In that case, the trade mark owner had registered in Class 5 in relation to ‘pharmaceutical preparations’. It had used its mark in relation to diagnostic testing preparations and, consequently, on an ordinary interpretation of the specification, it appeared that there had been no relevant use of the mark at all. However, Lloyd-Jones J held that the mark had in fact been used for a subset of the claimed goods in light of evidence that, for the purposes of the classification system, diagnostic preparations were a subset of pharmaceutical preparations.^94^

Using Nice as an interpretive aid offers a number of advantages. However, these advantages are only manifest if one accepts that the person consulting the register can and should be attributed with specialist knowledge. It must, nevertheless, be acknowledged that it has at times been argued that there are disadvantages to using Nice as an interpretive aid that go beyond the fact that it would be inconsistent with the trader-centric nature of the registration system. The key disadvantages of using Nice are said to be that: (i) the Nice Classification changes over time; (ii) the classes may not be mutually exclusive, and difficult borderline cases can and do occur; and (iii) decisions as to classification are for offices alone and are not subject to judicial oversight.^95^

Taking these alleged disadvantages in turn, the point about reclassification of goods and services following revisions to Nice is superficially compelling. It used to be the case, for example, that horseshoes were classified in Class 6 (metal goods), but in the latest version of Nice they are contained in Class 18 (clothing for animals). Keeping track of reclassified goods and services can be complex, and hence it might be thought that a better way of ensuring predictability and certainty is simply to set Nice aside. On closer examination, however, any move away from Nice causes far more problems than it solves. Knowing which version of Nice applies may be inconvenient, but by failing to draw on Nice, parties will close off a key source of guidance that can help identify and set the margins of otherwise broad terms.

Turning to the second objection to the use of Nice, concerns about the classes not being mutually exclusive and about difficult boundary cases are often exaggerated. Situations where goods can be classified in multiple classes are rare, arising mostly where goods are ‘multipurpose composite objects’ with multiple, equal functions. But the fact that Nice places ‘radio clocks’ in Class 9 and ‘clock radios’ in Class 14 provides a poor justification for casting Nice aside when seeking to interpret the vast majority of goods or services that are not of this kind. Further, many difficult cases of potential overlap or boundary drawing are likely to disappear on closer examination. Consider, for example, the relationship between ‘milk beverages flavoured with coffee’ in Class 29 and ‘coffee beverages with milk’ in Class 30 that we raised in section 2. The similarity in language naturally suggests that there is either an overlap here or a difficult boundary to be drawn. But on closer inspection, the problem disappears: ‘milk beverages flavoured with coffee’ refers to a subset of flavoured milk products that also includes strawberry milk, chocolate milk and the like; whereas ‘coffee beverages with milk’ refers to the range of beverages sold as coffee that contain milk or to which milk can be added (flat whites, cappuccinos, americanos with milk, etc). Nevertheless, even if the concern is sometimes exaggerated, overlaps and boundary problems can and do arise. To return to another example considered in section 2, there is almost certainly no clear demarcation between ‘tax planning’ in Class 35 and ‘financial advisory services related to tax’ in Class 36. Such cases may be relatively rare, but they do demand a response—the appropriate response being that developed by the General Court. As was seen above, the General Court has taken the view, qualifying the *Wenz* approach, that in cases of genuine uncertainty or overlap the trade mark owner has to be given the benefit of the doubt. Consequently, if the owner is a tax professional that can demonstrate use of its mark in relation to tax-related services, the tribunal can make a finding that the mark has been put to genuine use without trying to parse the difference between ‘tax planning’ and tax ‘advisory services’.

The third concern about the lack of judicial oversight can be illustrated by imagining a trade mark applicant who encounters a stubborn or incompetent trade mark examiner who insists that the applicant’s goods or services should be classified in a particular way. The concern is that because there is no mechanism by which office decisions as to classification can be challenged, an applicant might find itself forced to register in the wrong class.^96^ If claims are interpreted by reference to Nice, this could have a significant impact on the scope of protection, in some cases perhaps rendering the registration nugatory. Part of the answer is to ensure that any use of Nice as an aid to interpretation is subject to a manifest error standard, as it is in the EU. Where a mark has been registered in an obviously unsuitable class, the specification has to be taken at face value. A manifest error exception will protect not merely against office-induced errors, but also the more common problem of the applicant having filed in the wrong class and the office having failed to notice the problem (cases that, in any event, demand a less sympathetic treatment).^97^ A manifest error standard does, of course, leave applicants vulnerable to less obvious mistakes, that is, where language is more apt to describe goods falling into a different class, but is not clearly and obviously inappropriate in the class selected. In such cases, an applicant might be left with a very limited (possibly meaningless) sphere of protection. This is, however, a price that is worth paying, given the advantages of using Nice as an aid to interpretation.

We would therefore advocate for adoption of the *Wenz* approach to reading specifications, together with qualifications for cases of manifest error and genuine overlap. In doing so, we would also suggest that a willingness to embrace the idea that the hypothetical reader of the register can only ever be an expert in the field has other advantages beyond opening the door to using Nice as an aid to interpretation. One such advantage is that it would allow us to reconsider the rules governing the representation and interpretation of signs from first principles. It would allow us to recognise that the merits and demerits of, say, the ‘what you see is what you get’ approach need to be understood in terms of incentivising appropriate claiming on the part of applicants, with due regard being given to the fact that any change in approach creates significant transitional costs even for those versed in trade mark practice. Judged in this light, it might well still be concluded that a move to ‘what you see is what you get’ is desirable, but it would be a view grounded in appropriate considerations of policy. The ordinary trader test, in contrast, obscures these policy considerations. For instance, if the ‘black and white covers all approach’ facilitated registration in a form that was not accessible to the target audience of ordinary traders, then complaints about the retroactive effect of a change in practice have much less bite: such marks were in a sense never properly registered because they were never truly accessible to the target audience.

In some ways, however, the most important advantage of embracing an expert reader standard may be more amorphous. The idea that the register *can* be read by the ordinary trader is a precondition for concluding that the register *should* be read by the ordinary trader. As such, it is an idea that is enmeshed with questions as to whether, and if so to what extent, the ‘public’ nature of the register should weigh on the application of particular legal rules. In Australia, for example, it has been held that the adoption of a mark without conducting a search of the register is enough to impugn the motives of a later market entrant, precluding the operation of the ‘honest concurrent use’ exception to registration and defences to infringement that require a showing of ‘good faith’.^98^ Although the UK has taken a more nuanced approach to this issue in interpreting its ‘honest concurrent use’ and ‘honest practices in industrial or commercial matters’ provisions,^99^ it is an approach that still places a premium on the idea that traders ought to conduct searches.^100^ Such decisions rest on a vision of the accessibility of the register to ordinary traders that is at odds with what we know about awareness of the trade mark system among business communities.^101^ Our aim in raising this issue is not to suggest that incorrect outcomes have necessarily been reached. Rather, our point is simply that the idea that trade mark registers are repositories of information that are designed to be read by ordinary traders has the potential to weigh on how we approach a range of other important legal issues, and that this requires much closer critical attention.

## 5. Reading Specifications in the Age of AI

On two occasions I have been asked,—‘Pray, Mr Babbage, if you put into the machine wrong figures, will the right answers come out?’ In one case a member of the Upper, and in the other a member of the Lower, House put this question. I am not able rightly to apprehend the kind of confusion of ideas that could provoke such a question.^102^

Before concluding, it is necessary to say something about the impact of emerging technologies on the question of how we should conceive of the reader of the trade marks register. Trade mark offices are increasingly looking to AI-assisted search technologies to make automated assessments of ‘similarity’.^103^ A notable example is IP Australia’s ‘TM Checker’, which was formally launched in March 2024.^104^ This publicly available product allows a user to input its desired sign and specification, following which the user is provided with an automatically generated list of potentially conflicting marks or applications on the Australian register, an assessment made using AI-assisted algorithms. The UKIPO offers a similar product called ‘Trade Mark Pre-apply’,^105^ and it is proposed that the EUIPO’s ‘pre-assessment check’ tool will soon be able to check for conflicts with earlier marks.^106^ These services, which appear to have been designed primarily to assist small and medium enterprises,^107^ provide only high-level ‘pre-clearance’ information to potential applicants, and are presented as ‘guides’ without guarantees as to legal accuracy.^108^ Further, current search functionality is limited. For example, TM Checker only appears to search for ‘related’ classes of goods and services. This means, for example, that a search for MOTHER or MOTHERSKY for ‘coffee’ in Class 30 will not currently throw up a conflict with the earlier registered MOTHER in the unrelated Class 32 for ‘non-alcoholic beverages’, notwithstanding the findings in *Energy Beverages*. Despite these limitations, offices will continue to invest in and refine these AI-assisted search tools with a view to integrating them into their formal decision-making processes.^109^

The fact that registers are increasingly being read by ‘machines’ does not, however, make the issues we have explored in this article redundant. On the contrary, it is all the more important that we address the question of how information appearing on registers should be interpreted. This is because if human decision makers have failed to adopt clear, consistent and normatively desirable interpretive techniques, this will in turn minimise the value of the information that is subsequently generated by automated search tools.

To explain, all AI-assisted tools designed to search for similarities (either between signs, or between goods or services) need to be trained on datasets. Such data can only come from human-generated sources. TM Checker, for example, is trained on IP Australia’s internal examination data, being examiners’ notes on applications for registration, as well as ‘historical trade mark data’, which may well include decisions of hearing officers and courts.^110^ As these AI-assisted tools are developed over time to provide more sophisticated information on ‘similarities’, they will still need to be trained on the same human-generated datasets. What is of critical importance here is that the quality of the information that any AI-assisted search tool will be able to generate over time is a direct function of the quality of the information that is contained in these datasets. Looked at the other way, there is a risk that ‘biases in the training data will be unintentionally embedded in the training model[s] developed from that data’^111^ and, consequently, in the outputs generated from the use of those models.

The most obvious problem would appear to be where the dataset involves a group of decisions where tribunals have consistently misapplied the law. For example, if examiners and hearing officers have routinely made overbroad findings as to when two marks are ‘substantially identical’ with each other, contrary to authority, then these outcomes will likely be reflected in the results generated by AI-assisted searches for substantially identical marks.^112^ Another, more subtle problem is where the dataset involves decision makers adopting a normatively undesirable position in applying an established legal test. For example, it may be the case that examiners, in assessing whether two marks are ‘deceptively similar’, are routinely giving excessive weight to whether one mark is contained within another. Again, these biases are likely to be reproduced in any automatically generated results produced by an AI tool trained on such data.

Arguably, the most challenging problems arise when there is inconsistency within the dataset. Returning to the main themes of this article, this inconsistency might arise within registry-level decisions in the UK on whether goods or services are similar as to how far Nice is to be taken into account, given the ongoing uncertainty over the meaning of *Altecnic*. Inconsistency is even more likely to arise in Australia, where before *Energy Beverages* examiners would have taken Nice into account in interpreting the scope of specifications.^113^ The effect of *Energy Beverages* is that there must now be a subset of Australian decisions, finding that goods or services are dissimilar to each other, which are to be regarded as either wrong or at least of dubious value. Yet there would be no easy way of identifying, let alone extricating, these decisions from the dataset. Merely adding new decisions following *Energy Beverages* to the dataset, and even assigning these a higher ‘weighting’, would not ensure that an AI tool would make ‘similarity of goods or services’ assessments consistent with the new approach.

The point we wish to make is that if the issues we have raised in sections 2–4 are not addressed, there is a risk that this will leave in place decisions of both examiners and tribunals that are based on normatively undesirable principles and/or which are internally inconsistent. If these then form, and continue to form, the basis of training datasets, the automated decision-making tools on which both governments and the private sector^114^ increasingly wish to rely will merely serve to enshrine unsatisfactory practices. Of course, it will remain the case that bureaucratic decisions on ‘similarities’, even those made in reliance on AI, will be able to be challenged. But there would be something of an irony if this were to entrench a two-tiered trade mark system, where only those traders who are sufficiently well advised and well resourced would seek to challenge such decisions, with the remainder simply accepting the bureaucracy’s algorithmically-generated results, given that these new AI systems will purportedly benefit ordinary traders.

## 6. Conclusion

Lord Hoffmann once famously observed that a patent ‘specification is not a document *inter rusticos* for which broad allowances must be made’.^115^ In so doing, Lord Hoffmann was reminding us that patents are technical-legal-commercial constructs that have to be approached on the basis that they represent a considered statement of what the applicant is seeking to claim. They are to be interpreted against the background of shared understandings of matters such as how patent claims are structured. Registered trade marks are also not documents *inter rusticos*. But nor are they documents *inter mercatores*, as has too often been assumed. The very choice to have a system of trade mark protection based around registration counsels in favour of an expert reader standard, since it is not obvious why traders would know to use or consult the register without expert advice or education. Moreover, the rules governing conflicts between marks are such that the hypothetical trader will often need a lawyer on hand to explain what the information on the register means.^116^ Consequently, registered trade marks are documents best understood as being directed at trade mark insiders, who can interpret the likely bounds of protection thanks to a detailed understanding of the rules of infringement, who understand interpretive rules on how to interpret colour as a feature of a mark and who are perfectly capable of using Nice as an aid to interpretation. For members of this audience, and their automatons, coffee need not be, and, for reasons of clarity and predictability, ought not to be, a non-alcoholic beverage.

## Footnotes

1 Lon L Fuller, ‘Positivism and Fidelity to Law: A Reply to Professor Hart’ (1958) 71 Harv L Rev 630, 633.

2 cf Marc R Poirier, ‘On Whose Authority?: Linguists’ Claim of Expertise to Interpret Statutes’ (1995) 73 Wash U LQ 1025, 1033.

3 Pierre Schlag, ‘No Vehicles in the Park’ (1999) 23 Seattle U L Rev 381.

4 See section 2.

5 Nice Agreement Concerning the International Classification of Goods and Services for the Purposes of the Registration of Marks of 15 June 1957 (as amended on 28 September 1979).

6 Australia, the UK and all EU Member States apart from Cyprus and Malta are members of the Nice Special Union.

7 <https://nclpub.wipo.int/enfr/>. In the UK, Nice is the prescribed system of classification: Trade Marks Act 1994, s 34 and Trade Marks Rules 2008, rr 7–8. This is also the case in the EU: Directive (EU) 2015/2436 of the European Parliament and of the Council of 16 December 2015 to approximate the laws of the Member States relating to trade marks [2015] OJ L336/1 (EUTMD), art 39(1); Regulation (EU) 2017/1001 of the European Parliament and of the Council of 14 June 2017 on the European Union trade mark [2017] OJ L154/1 (EUTMR), art 33(1). In Australia, the Trade Marks Regulations 1995 (Cth) require goods and services to be specified in accordance with 45 listed classes that replicate the Nice classes and their headings (and which are amended when the Nice Classification is amended): regs 3.1, 4.4 and sch 1.

8 James Mellor and others, *Kerly’s Law of Trade Marks and Trade Names* (17th edn, Sweet & Maxwell 2023) [6-010].

9 This principle can be traced back at least as far as *The Australian Wine Importers Ltd* (1889) 41 Ch D 278, 291 (Ch) (‘Now for the purpose of deciding whether two sets of goods are of the same description we must not, as it appears to me, lay too much stress on the classification in the schedule to the rules. You find there goods of the same description in different classes, and goods of different descriptions in the same class’). This case remains the foundation of modern Australian law: *Reckitt & Coleman (Australia) Ltd v Boden* (1945) 70 CLR 84, 90 (Latham CJ). In the UK, see Trade Marks Act 1994, s 60A. In the EU, see EUTMD (n 7) art 39(7) and EUTMR (n 7) art 33(7).

10 Nice Agreement (n 5) art 2.

11 See WIPO, ‘Frequently Asked Questions: Nice Classification’ <www.wipo.int/classifications/nice/en/faq.html>.

12 See also *SkyKick UK Ltd v Sky Ltd* [2024] UKSC 36, [26]–[27] (Lord Kitchin). For a detailed study of the Nice classes and the administration of the Nice Classification, see Jessie N Roberts, *International Trademark Classification: A Guide to the Nice Agreement* (5th edn, OUP 2017).

13 <https://euipo.europa.eu/ec2/>.

14 <https://webaccess.wipo.int/mgs/>.

15 See IP Australia, ‘Classification Update’ (November 2023) <www.wipo.int/edocs/mdocs/madrid/en/mm_ld_wg_21/mm_ld_wg_21_roundtable_topic_3_australia.pdf>.

16 See IP Australia, ‘Adopting the Madrid Goods and Services List’ (6 January 2024) <www.ipaustralia.gov.au/news-and-community/news/adopting-the-madrid-goods-and-services-list> (explaining the decision of the Australian office to adopt the MGS list from March 2024).

17 Some jurisdictions also charge higher fees where specifications include terms that are outside approved lists: see eg Trade Marks Regulations 1995 (Cth), sch 9, item 1(a).

18 Offices have taken some *de minimis* steps to restrict the use of extremely broad language in specifications. For example, the EUIPO has determined that some terms previously accepted are unacceptably vague, such as ‘machines’ or ‘repair services’: see EUIPO Trade Mark Guidelines (2024), Part B, s 3, 4.3.1. However, other general terms, such as ‘computer software’, ‘vehicles’ and ‘clothing’, remain acceptable, despite the fact that each of these descriptions covers a broad array of subject matter segmented in the market by price, use and trade channel.

19
*Unilever Plc v Johnson Wax Ltd* [1989] 1 FSR 145, 155 (Ch).

20 See section 4.

21 [2023] FCAFC 44, (2023) 407 ALR 473.

22 [2024] FCAFC 149.

23
*Energy Beverages* (FCAFC) (n 21) [132] (our emphasis).

24 ibid [136].

25
*Energy Beverages LLC v Cantarella Bros Pty Ltd* [2022] FCA 113, (2022) 403 ALR 299 [314]. Having said this, the Full Court on appeal indicated that even if coffee beverages were not ‘non-alcoholic beverages’ within the meaning of the MOTHER specification, it would have been prepared to find that the two sets of goods were sufficiently similar: *Energy Beverages* (FCAFC) (n 21) [153].

26
*Killer Queen* (n 22) [57(4)].

27 ibid [57(5)].

28 ibid [59] (approving *Taylor v Killer Queen, LLC (No 5)* [2023] FCA 364, (2023) 172 IPR 1 [340]).

29
*Killer Queen* (n 22) [57(6)].

30 ibid [60]. The Court thereby explained away two seemingly inconsistent first instance decisions placing some interpretive weight on Nice, namely *Flexopack SA Plastics Industry v Flexopack Australia Pty Ltd* [2016] FCA 235, (2016) 118 IPR 239 [74] and *Trident Seafoods Corporation v Trident Foods Pty Ltd* [2018] FCA 1490, (2018) 137 IPR 65 [48].

31
*Killer Queen* (n 22) [60]. The Court went on to say: ‘primacy must be given to the words actually chosen by the trade mark owner to delimit the scope of the monopoly they claim. The question of the proper classification of the goods and services can only arise after the specification has been properly construed. Thus, it is the proper construction of the specification that informs the question of classification, not the other way around’.

32 [2003] FCA 816, (2003) AIPC ¶91-904 [43]–[44].

33 For an earlier case in which a similar approach was taken, see *Mem Co Inc v Cussons (International) Ltd* [1974] RPC 7, 28 (SC Bermuda).

34 See n 30 above. See also *Allergan Australia Pty Ltd v Self Care IP Holdings Pty Ltd* [2020] FCA 1530, (2020) 156 IPR 413 [273].

35
*Tornado Trade Mark* [1979] RPC 155, 159 (UK Registry) (‘apparatus (hand tools) for inserting or applying fasteners’ in Class 8 held not to extend to electrically operated hand tools, since motorised hand-held tools fell in Class 7); *Acetest Trade Mark* [1967] FSR 315 (Ch) (considering the relevance of Nice class headings in determining the meaning of the specified term ‘pharmaceutical preparations’. We return to this case in section 4).

36 EUIPO Guidelines (n 18) Part B, s 3, 4.2.2.

37 ibid (emphasis in original).

38 Case T-175/06 *Coca-Cola Co v OHIM* ECLI:EU:T:2008:212; Case T-221/12 *Sunrider Corporation v OHIM* ECLI:EU:T:2014:25; Case T‑199/13 *DTM Ricambi Srl v OHIM* ECLI:EU:T:2014:761; Case T‑367/16 *Brunner v EUIPO* ECLI:EU:T:2018:28; Case T‑89/17 *Erwin Müller GmbH v EUIPO* ECLI:EU:T:2018:353; Case T‑20/19 *Pablosky SL v EUIPO* ECLI:EU:T:2020:309; Case T‑588/19 *Novomatic AG v EUIPO* ECLI:EU:T:2021:157; Case T‑397/20 *Allergan Holdings France SAS v EUIPO* ECLI:EU:T:2021:653; Case T-794/21 *Wenz Kunststoff GmbH & Co KG v EUIPO* ECLI:EU:T:2023:211; Case T‑73/23 *Tertianum AG v EUIPO* ECLI:EU:T:2024:578.

39
*Wenz* (n 38).

40 ibid [21].

41 ibid [22]. The Court had made a similar point in earlier cases. See eg *Coca-Cola* (n 38) [74]–[75]; *Sunrider Corporation* (n 38) [31]–[32]; *Brunner* (n 38) [50]; *Erwin Müller* (n 38) [33]; *Pablosky* (n 38) [55]; *Novomatic* (n 38) [41].

42
*Wenz* (n 38) [23]. As authority for the first quoted sentence, the Court cited *DTM Ricambi* (n 38) [35]. As authority for the second quoted sentence, it cited *Allergan* (n 38) [35]. See also *Pablosky* (n 38) [55] and, most recently, *Tertianum* (n 38) [64]–[65].

43 See Case T-372/20 *Dermavita Company Sarl v EUIPO* ECLI:EU:T:2021:652 [55]; Case T-58/23 *Supermac’s (Holdings) Ltd v EUIPO* ECLI:EU:T:2024:360 [31]–[32]. Admittedly, the Court in *Supermac’s* also made some other general comments about the ‘solely’ administrative purpose of Nice ([30]) that could be read as precluding the use of Nice as an interpretive aid. However, these comments were not based on a properly reasoned or argued understanding of the issue under consideration here. Viewed through the eyes of a common lawyer, these comments can be dismissed as unhelpful dicta. Much the same can be said about earlier similar asides from the General Court in Case T-453/11 *Szajner v OHIM* ECLI:EU:T:2014:901 [88] and from the CJEU in Case C-400/14 P *Basic AG Lebensmittelhandel v OHIM* ECLI:EU:C:2015:514 [56]. In the latter case, for example, the CJEU emphasised the purely administrative role of Nice in the course of reaching the entirely uncontroversial conclusion that the distribution of foodstuffs and the wholesaling of foodstuffs are similar services. The CJEU does not, of course, recognise a formal distinction between ratio and obiter, but this does not mean that much weight needs to be given to introductory comments or mere asides. See further *Industrial Cleaning Equipment (Southampton) Ltd v Intelligent Cleaning Equipment Holdings Co Ltd* [2023] EWCA Civ 1451, [2024] FSR 3 [83] (Arnold LJ) (no great weight needs to be given to elements of a CJEU decision that ‘simply stated a bald conclusion’ and did not ‘contain … any analysis of the issue’). More generally, see Takis Tridimas, ‘Precedent and the Court of Justice: A Jurisprudence of Doubt?’ in Julie Dickson and Pavlos Eleftheriadis (eds), *Philosophical Foundations of European Union Law* (OUP 2012) 315 (arguing that an analogy can be drawn between the common law notion of obiter and mechanisms open to European courts for distinguishing the case at hand from earlier decisions).

44 See eg Case T-681/18 *ZAO Korporaciya ‘Masternet’ v EUIPO* ECLI:EU:T:2020:222.

45
*Wenz* (n 38) [25]. See also *DTM Ricambi* (n 38) [36].

46 [2001] EWCA Civ 1928, [2002] RPC 34.

47 ibid [22].

48 ibid [42].

49 [2010] EWHC 1211 (Ch), [2010] FSR 26 [75].

50 ibid.

51 [2019] EWHC 3357 (Ch), [2020] FSR 16.

52 ibid [84](g). See also *Senso-Rex Ltd v Gravity Products LLC* (O/557/21) [2021] ETMR 56 [30] (Appointed Person expressing support for this approach); *Swatch AG* (O/1041/23) [64] (hearing officer implicitly adopting this approach).

53 [2018] EWHC 3608 (Ch), [2019] FSR 8.

54 [2018] EWHC 1097 (Pat).

55
*Pathway IP* (n 53) [123]–[124]. See also *Montres Breguet SA v Samsung Electronics Co Ltd* [2022] EWHC 1127 (Ch), [2023] FSR 1 [132] (where Falk J left open whether ‘watches’ specified in Class 14 would extend to ‘smartwatches’). But cf *Swatch* (n 52) [64] as to the approach taken by the hearing officer in subsequent litigation between the same parties.

56
*FIL v Fidelis* (n 54) [90].

57 ibid [89].

58 See eg *British Sugar Plc v James Robertson & Sons Ltd* [1996] RPC 281, 289 (Ch); *Montres Breguet* (n 55) [120].

59
*FIL v Fidelis* (n 54) [86]; *Pathway IP* (n 53) [113].

60
*Multi-Access* (n 51) [84](g).

61 [2018] FCA 993, (2018) 358 ALR 336.

62 ibid [122].

63 The judicially developed rule prior to the Trade Marks Act 1995 (Cth) coming into force was different. See *William Charlick Ltd v Wilkinson & Co Pty Ltd* (1913) 16 CLR 370, 373 (Barton ACJ) (representation in colour creates an implied limitation).

64 See eg *Unilever* (n 19).

65 See Graeme B Dinwoodie and Dev S Gangjee, ‘The Image of the Consumer in EU Trade Mark Law’ in Dorota Leczykiewicz and Stephen Weatherill (eds), *The Images of the Consumer in EU Law: Legislation, Free Movement and Competition Law* (Hart Publishing 2015).

66 In the period before the CJEU’s decision in *Sieckmann* (discussed below), compare *British Sugar* (n 58) 289 (‘When it comes to construing a word used in a trade mark specification, one is concerned with how the product is, as a practical matter, regarded for the purposes of trade. After all a trade mark specification is concerned with use in trade’) with *Altecnic* (n 46) [42] (quoted above n 48).

67 Case C-273/00 ECLI:EU:C:2002:748 [51].

68 ibid [48].

69 First Council Directive 89/104/EEC to approximate the laws of the Member States relating to trade marks [1989] OJ L40/1, art 2. See now EUTMD (n 7) art 3(b).

70 Case C-307/10 ECLI:EU:C:2012:361.

71 ibid [48]–[49], [56].

72 See also Shane Creamer, ‘The IP Translator Case and Reform of Trade Mark Registration in Europe’ (2016) 11 JIPLP 929, 930.

73 European Trade Mark and Design Network, ‘Common Communication on the Common Practice of the Scope of Protection of Black and White (“B&W”) Marks’ (15 April 2014) <https://euipo.europa.eu/knowledge/course/view.php?id=1791&lang=de>.

74 Wenz’s appeal to the Court of Justice was discontinued: Case C-418/23 P *Wenz Kunststoff GmBH & Co KG v EUIPO* ECLI:EU:C:2023:931.

75 See n 43 above.

76 See EUTMD (n 7) art 39(5); EUTMR (n 7) art 33(5) (‘The use of general terms, including the general indications of the class headings of the Nice Classification, *shall be interpreted as including all the goods or services clearly covered by the literal meaning of the indication or term*’ (our emphasis)). Read in context, this provision was obviously intended to do no more than codify *IP Translator.* Moreover, the expression ‘clearly covered’ is sufficiently malleable to allow terms to be restricted by reference to Nice: Case T-47/23 *Jeleń v EUIPO* ECLI:EU:T:2024:12 [23]–[24] (claim for the ‘sale of clothing and footwear’ in Class 35 did not have to be read as encompassing the sale of clothing and footwear for animals given the manner in which Nice segregates these products, and art 33(5) of the EUTMR did not compel a different result). Nevertheless, for a court minded to overturn the *Wenz* line, these provisions could be read in a more expansive manner.

77 See *Industrial Cleaning Equipment* (n 43) [65], [85] (Arnold LJ), [122] (Nugee LJ) (Court of Appeal diverging from CJEU case law for the first time in a trade mark decision, in a case involving the relevant time for assessing acquiescence under s 48 of the Trade Marks Act 1994). In circumstances where conflict develops between a CJEU decision and later decisions of the General Court or EUIPO Boards of Appeal, this is likely to become a relevant factor that a higher UK court can take into account in determining whether to depart from that CJEU authority. See Retained EU Law (Revocation and Reform) Act 2023, s 6(3), which when in force will add a new s 6(5)(b) to the European Union (Withdrawal) Act 2018.

78
*Sieckmann* (n 67) [48], [51].

79 See Robert Burrell and Michael Handler, ‘(Re)claiming Trade Mark Protection’ in Graeme B Dinwoodie and Mark D Janis (eds), *Research Handbook on Trademark Law Reform* (Edward Elgar 2021) 63–64.

80 See Trade Marks Act 1995 (Cth), s 120(3); Trade Marks Act 1994 (UK), s 10(3); EUTMD (n 7) art 10(2)(c); EUTMR (n 7) art 9(2)(c).

81 See *Kerly* (n 8) [10-093]–[10-094].

82 See Wikipedia, ‘Cafeteria’ <https://en.wikipedia.org/wiki/Cafeteria>.

83 This data is derived from a search of IP Australia’s ‘Australian Trade Mark Search’ database <https://search.ipaustralia.gov.au/trademarks/search/advanced> on 1 October 2024, using the ‘Advanced Search’ function, and searching for these terms in the ‘Goods & services’ option for the dates selected.

84 See ibid for the methodology used to conduct this search.

85
*Multi-Access* (n 51) [90]. To much the same effect, see *GE Trade Mark* [1969] RPC 418, 458–59 (Ch).

86 cf *Avnet Inc v Isoact Ltd* [1998] FSR 16, 19 (Ch).

87 Classes 9 and 10 are not treated as ‘related’ for the purpose of office searching in either Australia (see IP Australia, *Trade Marks Office Manual of Practice and Procedure* pt 14 Annex A12 (20 April 2022) <https://manuals.ipaustralia.gov.au/trademark/annex-a12---cross-search-classes-from-january-2015>) or the UK (see UKIPO, ‘Trade Marks Cross Search List’ (26 July 2019) <www.gov.uk/government/publications/searching-for-similar-trade-mark-goodsservices-in-other-classes/trade-mark-cross-search-list>).

88 Trade Marks Act 1995 (Cth), s 92(4)(b) (prescribing a three-year period); Trade Marks Act 1994 (UK), s 46(1); EUTMD (n 7) art 16(1); EUTMR (n 7) art 18(1) (each prescribing five-year periods).

89 Richard A Posner and William M Landes, ‘The Economics of Trademark Law’ (1988) 78 Trademark Rep 267.

90 In Australia, see *Hills Industries Ltd v Bitek Pty Ltd* [2011] FCA 94, (2011) 214 FCR 396 [301]. In the UK, see *Stichting BDO v BDO Unibank Inc* [2013] EWHC 418 (Ch), [2013] FSR 35 [54]–[58]. In the EU, see Case T-126/03 *Reckitt Benckiser (España) SL v OHIM* ECLI:EU:T:2005:288 [45]–[46]; Joined Cases C-720/18 and C-721/18 *Ferrari SpA v DU* ECLI:EU:C:2020:854 [41].

91 See Trade Marks Act 1994, s 34(2) (Registrar’s decision as to classification ‘shall be final’); Trade Marks Act 1995 (Cth), s 32(b) (classification decisions ‘not subject to appeal and may not be called into question in an appeal or other proceedings under this Act’).

92 See Dev S Gangjee, ‘Paying the Price for Admission: Non-Traditional Marks across Registration and Enforcement’ in Irene Calboli and Martin Senftleben (eds), *The Protection of Non-Traditional Trademarks: Critical Perspectives* (OUP 2018).

93 In this regard, *Energy Beverages* can be seen as a real-life illustration of the problem identified in *Multi-Access* (n 51) [85] (posing the hypothetical of a manufacturer of valves for babies’ bottles who owns a registration for ‘valves’ using its registration to extract rents from manufacturers of products containing valves that fall into different Nice classes).

94
*Acetest* (n 35) 317–18.

95 See also *Pathway IP* (n 53) [76].

96 See Trade Marks Act 1995 (Cth), s 32; Trade Marks Act 1994 (UK), s 34(2).

97 This seems to have been what occurred in the three ‘manifest error’ cases discussed in section 2: *Nikken Wellness* (n 32), *Dermavita* (n 43) and *Supermac’s* (n 43).

98 On ‘honest concurrent use’, see eg *Insight Radiology Pty Ltd v Insight Clinical Imaging Pty Ltd* [2016] FCA 1406, (2016) 122 IPR 232 [125]; *Pham Global Pty Ltd v Insight Clinical Imaging Pty Ltd* [2017] FCAFC 83, (2017) 251 FCR 379 [103]. On ‘good faith’ for the purposes of the defences to infringement, see eg *Flexopack* (n 30) [107]–[118]; *Hemmes Trading Pty Ltd v Establishment 203 Pty Ltd* [2024] FCA 1100, (2024) 179 IPR 315 [65].

99 See eg *Hotel Cipriani Srl v Cipriani (Grosvenor Street) Ltd* [2009] EWHC 3032 (Ch), [2009] RPC 9 [150]–[151]; *Samuel Smith Old Brewery (Tadcaster) v Lee* [2011] EWHC 1879 (Ch), [2012] FSR 7 [118](i), [124], [132]; *Maier v Asos Plc* [2015] EWCA Civ 220, [2015] FSR 20 [159] (Kitchin LJ).

100 See eg *GNAT and Co Ltd v West Lake East Ltd* [2022] EWHC 319 (IPEC) [79]–[88].

101 See Advisory Council on Intellectual Property, *A Review of the Relationship between Trade Marks and Business Names, Company Names and Domain Names* (March 2006) 1, 27, 29–30; Small Business Connections, ‘Trademarks Are Critical Assets—So Why Do Only 4% of SMEs Have Them?’ (1 February 2023) <https://smallbusinessconnections.com.au/trade-marks-are-critical-assets-so-why-do-only-4-of-smes-have-them/>.

102 Charles Babbage, *Passages from the Life of a Philosopher* (Longman, Green, Longman, Roberts and Green 1864) 67.

103 See Dev S Gangjee, ‘Eye, Robot: Artificial Intelligence and Trade Mark Registers’ in Niklas Bruun and others (eds), *Transition and Coherence in Intellectual Property Law: Essays in Honour of Annette Kur* (CUP 2021) 178–84; Anke Moerland and Conrado Freitas, ‘Artificial Intelligence and Trade Mark Assessment’ in Jyh-An Lee, Reto Hilty and Kung-Chung Liu (eds), *Artificial Intelligence & Intellectual Property* (OUP 2021); Ángela Martínez López, ‘An Artificial Intelligence Toolkit for Future-proof Trade Mark Offices across the Globe’ [2024] EIPR 382.

104 <https://tmchecker.ipaustralia.gov.au/start>.

105 See Intellectual Property Office, ‘Introducing the Trade Mark Pre-apply Service’ (29 October 2020) <https://ipo.blog.gov.uk/2020/10/29/introducing-the-trade-mark-pre-apply-service/>.

106 See EUIPO, ‘New Pre-assessment Checks in Your EUTM Application’ (2 June 2023) <https://euipo.europa.eu/ohimportal/en/-/news/new-pre-assessment-checks-in-your-eutm-application>.

107 IP Australia, ‘Australian IP Report 2024’ <www.ipaustralia.gov.au/tools-and-research/professional-resources/data-research-and-reports/australian-ip-report-2024>, 54.

108 See IP Australia, ‘TM Checker: About This Site: Disclaimer’ <https://tmchecker.ipaustralia.gov.au/about?open=privacy>.

109 See eg EUIPO, *2023 Consolidated Annual Activity Report* <https://euipo.europa.eu/tunnel-web/secure/webdav/guest/document_library/contentPdfs/about_euipo/annual_report/Annual_Activity_Report_2023_en.pdf> 19 (on the EUIPO’s internal use of its AI-assisted ‘G&S Comparer’ tool).

110 See IP Australia, ‘TM Checker: About This Site’ <https://tmchecker.ipaustralia.gov.au/about?open=privacy>.

111 Dev S Gangjee, ‘A Quotidian Revolution: Artificial Intelligence and Trade Mark Law’ in Ryan Abbott (ed), *Research Handbook on Intellectual Property and Artificial Intelligence* (Edward Elgar 2022) 338.

112 See Michael Handler, ‘Trade Mark Law’s Identity Crisis (Part 1)’ (2021) 44 UNSWLJ 394.

113 See nn 30 and 34 above.

114 See generally Sonia K Katyal and Aniket Kesari, ‘Trademark Search, Artificial Intelligence and the Role of the Private Sector’ (2021) 35 Berkeley Tech LJ 501.

115
*Kirin-Amgen Inc v Hoechst Marion Roussel Ltd* [2004] UKHL 46, [2005] RPC 9 [34].

116 Our thanks go to Rebecca Tushnet for pushing us on this point.

